# Cement Paste Mixture Proportioning with Particle Packing Theory: An Ambiguous Effect of Microsilica

**DOI:** 10.3390/ma14226970

**Published:** 2021-11-18

**Authors:** Paweł Niewiadomski, Anna Karolak, Damian Stefaniuk, Aleksandra Królicka, Jacek Szymanowski, Łukasz Sadowski

**Affiliations:** 1 Faculty of Civil Engineering, Wroclaw University of Science and Technology, Wybrzeże Wyspiańskiego 27, 50-370 Wroclaw, Poland; anna.karolak@pwr.edu.pl (A.K.); damian.stefaniuk@pwr.edu.pl (D.S.); jacek.szymanowski@pwr.edu.pl (J.S.); lukasz.sadowski@pwr.edu.pl (Ł.S.); 2 Civil and Environmental Engineering Department, Cullen College of Engineering, University of Houston, 4726 Calhoun Road, Houston, TX 77204, USA; 3 Department of Metal Forming, Welding and Metrology, Wroclaw University of Science and Technology, Wybrzeże Wyspiańskiego 27, 50-370 Wroclaw, Poland; aleksandra.krolicka@pwr.edu.pl

**Keywords:** packing density model, microsilica, nanoparticles, cement paste, physical properties, mechanical properties

## Abstract

Recently, the research of innovative building materials is focused on applying supplementary materials in the form of micro- and nanopowders in cementitious composites due to the growing insistence on sustainable development. Considering above, in paper, a research on the effect of microsilica and SiO_2_ nanoparticles addition to cement paste, designed with Andreasen and Andersen (AA) packing density model (PDM), in terms of its physical and mechanical properties was conducted. Density, porosity, compressive strength, hardness, and modulus of indentation were investigated and compared regarding different amount of additives used in cement paste mixes. Microstructure of the obtained pastes was analyzed. The possibility of negative influence of alkali-silica reaction (ASR) on the mechanical properties of the obtained composites was analyzed. The results of the conducted investigations were discussed, and conclusions, also practical, were presented. The obtained results confirmed that the applied PDM may be an effective tool in cement paste design, when low porosity of prepared composite is required. On the other hand, the application of AA model did not bring satisfactory results of mechanical performance as expected, what was related, as shown by SEM imaging, with inhomogeneous dispersion of microsilica, and creation of agglomerates acting as reactive aggregates, what as a consequence caused ASR reaction, crack occurrence and lowered mechanical properties. Finally, the study found that the use of about 7.5% wt. of microsilica is the optimum in regards to obtain low porosity, while, to achieve improved mechanical properties, the use of 4 wt. % of microsilica seems to be optimal, in the case of tested cement pastes.

## 1. Introduction

In recent years, due to the inclination to join the trend of sustainable development, supplementary cementitious materials (SCM) have been commonly used in cement and concrete industry [1]. They can be added either separately or in blended cements. They are used in order to reduce the environmental pollution that is caused by the storage of by-products of many industrial processes as well as to obtain improved mechanical properties of concrete.

One of the SCM investigated and applied over time is microsilica, also called silica fume (SF). It is a finely divided residue, a waste product from ferro-alloy silicon industry. The reaction mechanism of silica fume blended with Portland cement leads to the production of a significant amount of calcium-silicate-hydrate (C-S-H) phase in hardened cementitious composites, which is associated with the strength in cement-based materials [2]. At the same time, silica fume acts as a micro-filler of the cement composites structure. According to numerous research conducted all over the world, e.g., [3], cement composites with the addition of the microsilica have several advantages, such as the following: increased strength parameters (compressive, tensile, and flexural strength), elastic modulus, toughness, as well as early compressive strength. Microsilica application brings benefits like higher durability, improved interfacial transition zone between cementitious matrix and aggregate, and bond strength [4]. Composites obtained with the use of microsilica are characterized by increased impermeability to water and chloride penetration, abrasion resistance as well as resistance to deicing salts, and chemical attack. Moreover, the possibility of replacing ordinary Portland cement with silica fume may be considered a step towards sustainable development in the civil engineering industry and a way to decrease environmental problems [5,6].

Another ultra-fine additive with a maximum grain size of 100 nm, considered in cement industry applications, are nanoparticles. According to [7], improving the composition of concrete with the use of additives with increasingly smaller grain size has a positive effect on the material structure. The mechanism describing the positive impact of nanoparticles on cement composites is as follows [8]: nanoparticles, that are the nuclei gathering around the products of the cement hydration due to their high chemical reactivity, play the role of a nano-filler for the material structure. Moreover, in the case of SiO_2_ and Al_2_O_3_ they react with calcium hydroxide and create additional amounts of C-S-H phase in the concrete structure. Therefore, they positively affect the physical properties of concrete by reducing porosity [9] and permeability [10]. Additionally, nanoparticles may improve the mechanical and functional parameters of the material [11]. In [12], it was proven that the addition of a certain amount of nanoparticles of SiO_2_ increases mechanical parameters of concrete such as compressive strength and flexural strength.

Finding the optimal amount of microsilica and nanoparticles addition to the concrete mixtures in order to obtain most favorable properties (especially compressive strength) was the aim of several studies [13,14,15,16,17]. Different approaches were presented in the literature, such as the following: experimental, analytical, computational, or multilevel ones [13,14]. Modern techniques based on artificial neural networks [15] or biogeography-based optimization [16] were undertaken to try to optimize the mixture composition of different concrete types. Based on the literature review, it can be concluded that the content of microsilica in cementitious materials varies from 1.0 to 30.0 wt.%, nevertheless the recommended amount appears to be in the range from 5.0 to 15.0 wt.% [17]. At the same time, a dosage of nanoparticles varies from 0.5 to 12.0 wt.%; however, the optimum range appears to be between 1.0 and 4.0 wt.% [18]. In both situations amount of additive depends on the type of base material (cement paste, mortar, concrete) and the aim of the modification. Despite many research in the subject, the question about the optimal amount of microsilica and nanoparticles in cement matrix and concrete mixture has not been answered properly yet. Many researchers, as a conclusion from their studies, claim that the issue of concrete mix design determination is still one of the most challenging tasks for scientists and engineers for the coming years.

In order to determine the optimal composition of the cementitious system, one of the packing density models may be used. Their main concept is based on increasing the amount of solids in unit volume in order to decrease the porosity and increase the mechanical properties of the cement composites [19]. One of the most commonly used packing density models is the modified Andreasen and Andersen (AA) model [20] and its basis was clearly presented in the paper [21]. The AA model was successfully used in work [22], where it was proven that flowability, packing density, and compressive strength may be positively affected in case of cement paste when AA model is applied. The modified Andreasen and Andersen model was used with positive effect as well, e.g., in the development of cement-based lightweight [23] and ultra-lightweight [24] composites maximizing the packing of lightweight fillers in cementitious systems. The modified AA model also proved its performance to design of self-compacting concrete [25], gypsum-based composites [26] or high-performance concrete [27].

An important issue in the modification of cement composites with additives containing silica is the alkali-silica reaction (ASR). ASR is caused by the reaction between the reactive amorphous silica, which is present in aggregate, and the highly alkaline solution of the cement paste. Besides excessive expansion, ASR reaction can result in cracking and microstructure degradation of cement composite, what was described among others in [28,29,30]. There are some methods preventing from ASR described in the literature that are based on applying appropriate additives, e.g., supplementary cementitious materials (SCM) like granulated blast furnace slag, fly ash, metakaolin [31], or metal ions [32]. Moreover, it is well known that microsilica is assumed as a very effective additive to mitigate ASR. Due to the fact that microsilica refines the pore structure and retards the transportation of alkalis to the reaction sites in a cementitious system with the reactive aggregates, it is known as an adequate material for ASR control [33]. However, some researchers reported that using microsilica in cement-based materials may lead to undesirable effects of worsening the structure of hardened composite, e.g., Ref. [34]. One of the reasons of that fact is that silica fume agglomerates or large siliceous particles exist and behave as small aggregates, rather than as pozzolans, which leads to an expansive reaction with alkalis in cement [35].

Taking the above into consideration, the aim of the study was to determine the optimal amount of microsilica and SiO_2_ nanoparticles using Andreasen and Andersen packing density model to improve selected physical and mechanical properties, such as porosity, compressive strength, and hardness of the obtained cement paste. Another aim of the work was to find the answer to whether the Andreasen and Andersen packing density model can be effectively applied at cement paste level. The scope of the study covered preparation of cement paste mixes, including reference one and mixtures with optimized amount of only microsilica and both microsilica and nanoparticles. The optimization procedure was based on particle size distribution (PSD) of each component of cement paste and consideration of the fine particles’ influence on the particle packing skeleton. The research included the following properties: density, porosity, compressive strength, hardness, and microstructure analysis. As microsilica and SiO_2_ nanoparticles were added to the cement paste mixture, the possibility of negative effects of the alkali-silica reaction was investigated as well.

**Figure 1:**
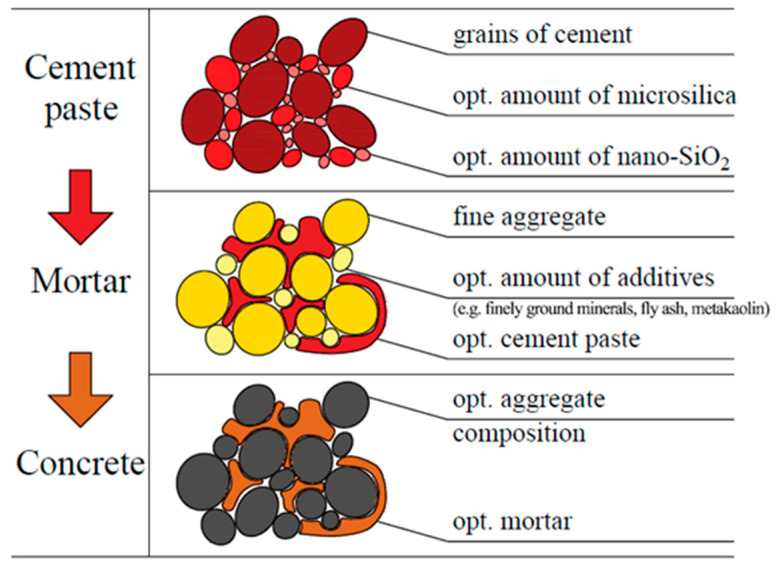
Cement-based materials design approach based on Andreasen and Andersen packing density model and concerning obtainment of the optimal composition, first of the paste, then of the mortar, and finally of the concrete.

Considering the fact that concrete is a complex structure built by components and additives with various grain sizes and the fact that its hardened form is characterized by diverse pore sizes, a question arises: what should be the optimal content of multi-size additives such as microsilica and nanoparticles to obtain composite with most fitted structure in regard to its properties? Moreover, does the use of modified Andreasen and Andersen packing density model provide enhancement to physical and mechanical properties of cement paste with addition of microsilica and nanoparticles? As an attempt to answer those questions, a research study on the effect of microsilica and nanoparticles’ addition to cement paste designed with AA model in terms of its physical and mechanical properties was conducted and presented in this paper. The paper should fill existing gap in the literature in the subject of optimization of cement-based material at the paste level. The presented work is the first step towards a general methodology of cement-based materials design approach, where at the beginning cement paste is optimized with the use of Andreasen and Andersen packing density model, which helps to properly design cement mortar and in the last stage concrete, what schematically was presented in Figure 1. Also, in the research conducted and presented in the paper, an unexpected and ambiguous effect of using microsilica in cement pastes is observed, which is worth sharing among the scientific environment.

## 2. Materials and Methods

### 2.1. Materials

**Figure 2:**
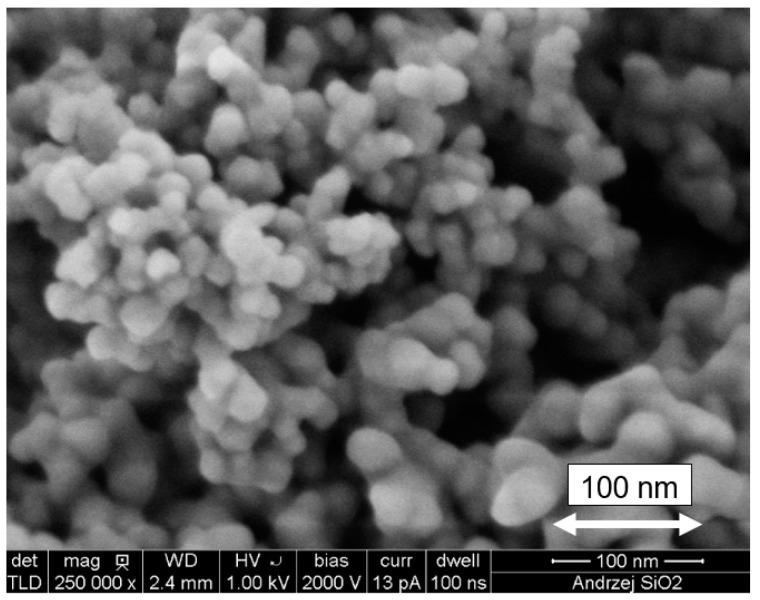
SEM image of SiO_2_ nanoparticles.

The following components were used to prepare cement pastes for investigations:

- Portland cement CEM I 42.5 R, commercially available and manufactured by Lafarge Cement SA (28-366 Małogoszcz, Poland), that meets the standard requirements of PN-EN 197-1 [36], with a density of 3.09 g/cm^3^, the specific surface area of 3360 cm^2^/g, and the chemical and phase composition, that was delivered by the manufacturer, shown in Table 1;

- microsilica 920 EN D, commercially available and manufactured by Elkem Silicones France (69486 Lyon, France), which meets the requirements of PN-EN 13263 [37], with a density of 1.76 g/cm^3^, the specific surface area of 15 ÷ 35 m^2^/g, pozzolanic activity index of minimum 100%, and has the chemical composition, that was delivered by the manufacturer, shown in Table 2;

- nanoparticles of SiO_2_ in the form of powder, delivered by Sigma-Aldrich company (61-626 Poznań, Poland), with a particle size of 10 ÷ 20 nm, a density at 25 °C of 2.4 g/cm^3^, a specific surface area of 450 m^2^/g and a purity of 99.5% (the SEM image of the used nanoparticles is shown in Figure 2);

- universal superplasticizer (SP) Sikament FM6 produced by Sika AG (CH-6341 Baar, Switzerland), based on sulfonated naphthalene and melamine polycondensates, with a density of 1.15 g/cm^3^.

**Figure 3:**
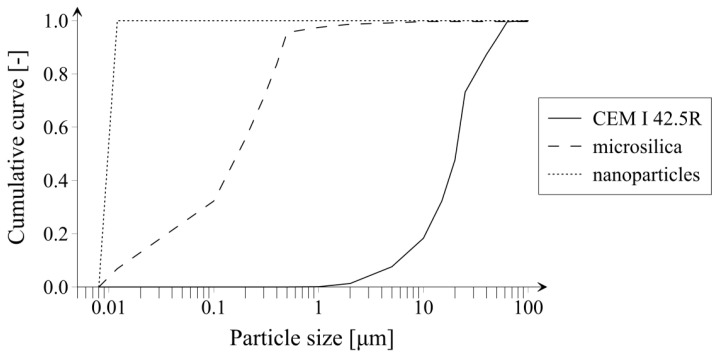
Particle size distribution of cement CEM I 42.5R, microsilica and nanoparticles.

**Figure 4:**
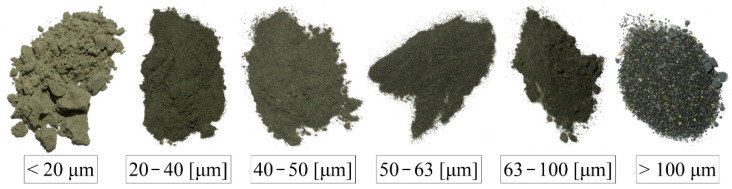
Particle size distribution of cement CEM I 42.5R, microsilica, and nanoparticles.

Below, in Figure 3, particle size distribution of cement, microsilica, and nanoparticles, which were applied in tests, is presented. For achieving a precise PSD, before performing sieve analysis, all constituents were dried at the temperature of 70 °C. In order to obtain PSD of cement, a sieve analysis was performed using sieves with mesh sizes of 100 µm, 63 µm, 50 µm, 40 µm, and 20 µm. As a result, cement grains of different sizes were separated, which can be seen in Figure 4. In case of microsilica, PSD was delivered by manufacturer, and in the case of nanoparticles, a linear particle size distribution was assumed.

### 2.2. Mix Design

Five different cement paste mixes were designed and prepared with the described components. One mix was made without the addition of microsilica nor nanoparticles, as a reference mix. The next two were made with a suitably low and high content of microsilica, as comparative mixtures. The last two cement paste mixes were prepared using the AA packing density model, whereof one was modified only with microsilica, while the other with both microsilica and nanoparticles. Water to binder (W/B) ratio was assumed to be equal to 0.36 for each mix, where B is the sum of weights of cement, microsilica, and nanoparticles. The volume of prepared mixes was fixed to be equal to 2.0 L. The amount of used superplasticizer was equal to 1.0% of cement weight, based on the manufacturer’s recommendations and author’s previous experience.

Different models were postulated by Larrard and Sedran [19,38] to design cementitious composites mixes, e.g., the solid suspension model, linear packing density model, or compressive packing model. However, in order to consider the influence of fine particles on the particle packing skeleton, the modified Andreasen and Andersen Equation (1) was proposed and herein used since very fine particles were considered in this study [39]:

P_t_(φ) = (φ^q^ − φ_min_^q^)/(φ_max_^q^ − φ_min_^q^), (1)

where P_t_(φ) is the fraction of all solids smaller than the particle size (φ) and φ_min_ and φ_max_ are the minimum and maximum particles size, respectively. Parameter q is the distribution modulus, and in this work the value of 0.28 was assumed, which is in the optimal range according to [20]. Equation (1) acts as a target function in order to optimize the granular materials composition. Therefore, the mix design is based on finding the experimental function that fits the target function as close as possible. For this purpose, the least-squares method was used. The target function used in the research was as follows:

(2) $$\mathrm{TF}=\sum_{i=1}^{n}{\left(P_{\exp}\left(\varphi_{i}^{i+1}\right)-P_{t}\left(\varphi_{i}^{i+1}\right)\right)}^{2},$$

where P_t_(φ) is evaluated based on Equation (1) and P_exp_(φ) is obtained in the experimental laboratory measurements.

### 2.3. Mixing Process and Samples Preparations

All mixtures were prepared in batches of 2.0 L volume, using an automatic mixture in accordance with PN-EN 196-1 [40]. The normal rotation speed of the mixer was constant and set to be equal to 140 rpm. Some trial and error tests were used to determine the optimal mixing procedure sequences and time considering the flowability of the developed cement pastes. The mixing process consisted of three stages. First, water with superplasticizer was stirred for 30 s. Next, cement, or cement previously manually mixed with microsilica for 30 s, was added and all was stirred for 90 s. Then, a fresh paste was mixed manually for 30 s in term to scrap some adhering materials from the bowl, and in the last stage all was stirred for another 60 s using double rotation speed. In the case of series CI_nSi, nanoparticles were first stirred together with water and superplasticizer for 60 s to provide uniform dispersion and prevent the formation of agglomerates, and additionally, ultrasonication was applied for 10 s after nanoparticles were stirred with water and superplasticizer. Finally, after casting and molding of specimens, the vibration table was used for 5 s to densify all cement paste mixes.

Four types of samples were prepared: cylindrical samples with a diameter of 17 mm and height of 34 mm designated for physical (density and porosity) and compressive strength tests, prismatic samples with the dimensions of 10 mm × 10 mm × 60 mm designated for ASR test, cylindrical samples with the diameter of 17 mm and height of 5 mm for indentation measurements and cubic samples with the dimensions of 10 mm × 10 mm × 10 mm designated for SEM analysis.

In order to get the surface of the samples properly prepared for the indentation tests, the surface of cylindrical samples was ground with the use of 320 grit sandpaper and then with the use of 9 μm, 3 μm and 1 μm graded diamond slurry until obtaining a demanded smooth surface.

The preparation process of samples intended for microstructure analysis involved immersing cubic samples with dimensions of 10 mm × 10 mm × 10 mm in a non-conductive epoxy resin, and then surface grounding by sandpaper (number in range from 320 to 1200), and polishing by diamond colloid with a size of diamonds in range from 6 μm to 1 μm. The electrical conductivity and thus the prevention of electrical charging was ensured by a conductive path formed of copper tape. Furthermore, prepared samples were coated with amorphous carbon with a thickness of 40 nm. The applied conductive coating was formed in a vacuum of 2 × 10^−6^ Torr (2 × 10^−5^ Pa) by the thermal sputtering method using graphite electrodes.

**Figure 5:**
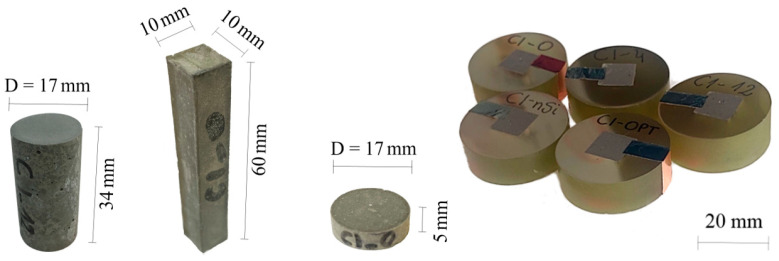
Types of samples used in tests.

All types of samples with their dimensions used in tests are presented in Figure 5.

### 2.4. Physical Properties

Tests of the basic physical properties of hardened cement pastes were made after 28 days of samples maturation. The density, bulk density, and porosity of each series were determined based on standard methods [41,42]. Fresh paste density was determined during casting as well.

Solid density (ρ_s_) of cement paste was determined according to the standard procedure [41] on samples of finely crushed material in a Le Chatelier flask and calculated according to the Formula (3):

ρ_s_ = m_s_/V_s_ [g/cm^3^], (3)

where: m_s_ means the mass of the solids [g], V_s_ means the volume of the solids [cm^3^].

Bulk density (ρ_o_) was determined according to [42] and calculated according to the Formula (4):

ρ_o_ = m/V [g/cm^3^], (4)

where: m_o_ means the mass of the sample dried at 105 °C [g], V means the volume of the sample [cm^3^].

Volume (V) of the sample was determined using the hydrostatic weighing method and calculated according to the Formula (5):

V = m_sat_ − m_h_/ρ_H2O_ [cm^3^], (5)

where: m_sat_ means the mass of the sample saturated with water [g], m_h_ means the mass of the sample on the hydrostatic weight [g], ρ_H2O_ means the density of water [g/cm^3^].

Total porosity (p) and solidity (s) of cement paste were calculated based on values of solid density and bulk density according to the following Formulas (6) and (7):

s = ρ_o_/_s_ [%], (6)

p = 1 − s [%].(7)

### 2.5. Mechanical Properties

In order to determine compressive strength (f_c_) at least 6 cylindrical samples with a diameter of 17 mm and height of 34 mm were tested. Compressive strength was investigated after 180 days of samples maturing, as the authors intention was to eliminate the effect of microsilica on the early compressive strength of cement paste. Until the time of testing, all samples were cured in a climate chamber at a temperature of 20 °C (±1 °C) and 95% R.H. (±5%).

### 2.6. Micromechanical Properties

Mechanical properties in microscale were evaluated using indentation technique, with samples matured 180 days, similarly to compressive strength tests. In brief, the method consists of pressing a diamond tip (Berkovich tip in this case) into a sample. With increasing load (F) and recording the depth (h) the F-h curve is derived. Then, analyzing the F-h curve, one can obtain the indentation modulus (M) from the Formula (8) according to [43]:

M = √π/2 × S/√A,(8)

and hardness (H) from the Formula (9):

H = F_max_/A, (9)

where S is the slope of the unloading part of the F-h curve, F_max_ is the maximum applied load, and A is the projected area of contact between the sample surface and indenter tip [44].

In this work, microindenter TTX-NHT (Anton Paar GmbH, Graz, Austria) was used. For each sample, 60 to 80 indentation tests were performed at random locations. The standard procedure was applied using one loading-unloading cycle (both loading and unloading part lasted 30 s) up to the maximum force of 5 N.

### 2.7. Microstructural Analysis

Microstructure investigations were carried out using the JSM-6610A (JEOL, Tokyo, Japan) Scanning Electron Microscope (SEM) equipped with a conventional tungsten filament. The microstructure of the samples was observed in the material contrast mode using back-scattered electrons (BSE) detector. An accelerating voltage of 20 kV, a beam current of 38 nA, and a working distance of 10 mm were utilized.

The analysis of chemical elements composition was performed using an energy-dispersed X-ray spectrometer (EDX, EDS) JEOL JED-2300 (JEOL, Tokyo, Japan). The count rate was set to approximately 4500 cps, and the dead time of the detector did not exceed 10. Quantitative analyses of measured chemical composition were determined using JEOL software (JEOL JED-2300 Analysis Station, Tokyo, Japan) with consideration of the ZAF method. Elementary mappings were determined in order to the relative chemical composition distribution visualization of the tested samples. The data acquisition time of elementary mapping was about 1 h for each investigated area. The intensity maps of the chemical elements were characterized by a resolution of 512 × 384 points with the observation field size of 370 μm × 277 μm.

### 2.8. ASR

Measurements of the samples’ elongation caused by possible ASR were conducted in this work. For this purpose, prisms with the dimensions of 10 mm × 10 mm × 60 mm were first cured in water for 24 h, and then placed in a hot water bath at 80 °C for another 24 h to gain reference length. In the next step, the specimens were stored in a tank containing 1 M NaOH solution at 80 °C for 14 days. The elongation was measured in the first and the last day during the 14 days testing period using a digital micrometer (1 μm accuracy). For each series, four samples in two directions were measured and the average value of elongation was reported.

## 3. Results, Analysis and Discussion

### 3.1. Mix Design

**Figure 6:**
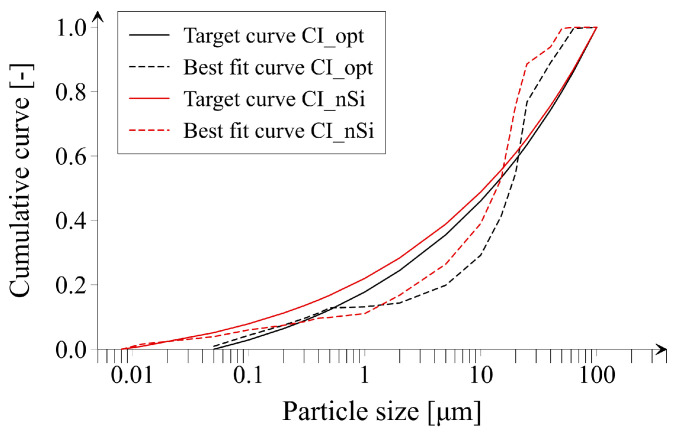
Particles size distribution of optimized cement paste mixes.

Figure 6 presents particles size distribution of optimized cement paste mixes CI_opt and CI_nSi obtained using Equation (2). In turn, the composition of all prepared cement paste mixes is shown Table 3. Considering rheological behavior of tested cement paste mixes, it should be noted that each of the mixtures was characterized by comparable workability assessed with the flow table test.

The literature presents an attempt to use a similar methodology to design lightweight cementitious composites with the addition of waste glass from the point of view of low density and thermal conductivity [23]. To obtain the optimal target grading curve the modified Andreasen and Andersen model was used. It has been shown that this model can be used successfully and that apart from obtaining a low thermal conductivity, the designed composites also had sufficient mechanical parameters.

### 3.2. Physical Properties

**Figure 7:**
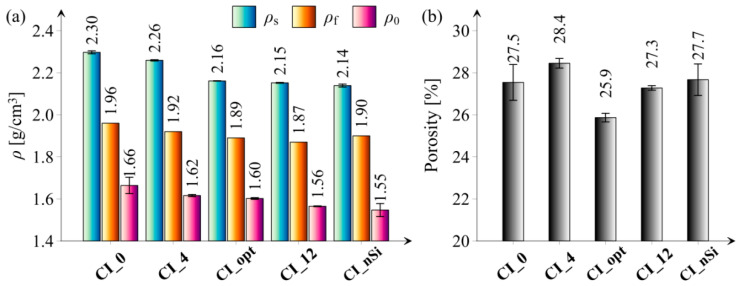
Results of physical properties of hardened cement paste of all tested series: (**a**) specific density (ρ_s_), fresh paste density (ρ_f_), and bulk density (ρ_o_), (**b**) porosity. Vertical bars indicate one standard deviation.

Figure 7 presents the results of specific density, fresh paste density, and bulk density as well as porosity of hardened cement paste of all tested series.

Analyzing the presented figures, it can be noticed that with the increasing amount of the microsilica additive (series CI_0, CI_4, CI_opt and CI_12), both the bulk density and the specific density decrease, which is an expected phenomenon considering the density of cement and microsilica. Still, it is also worth taking into account that by using microsilica as a partial replacement for cement, the weight of the cement-based composite is reduced (density of microsilica is equal to about 1.8 g/cm^3^ while the density of cement is equal to about 3.1 g/cm^3^). This fact can have a practical application, e.g., in the production of lightweight roof girders. What is more, the CI_opt series is characterized with the lowest porosity value among all the tested series. This fact may prove effectiveness of the AA packing density model used in the optimization process of cement paste mixes. What is more, obtained results confirmed conclusions of the research presented in [21], where extensive review on an application of Andreassen and Modified Andreassen Model on cementitious mixture design was performed. It should also be noted that the combination of the effect of microsilica and nanoparticles did not bring the expected effect in the form of obtaining the lowest porosity. The explanation for this phenomenon may be the large specific surface area of the used nanoparticles, which is related to the higher consumption of batch water and, consequently, obtains a worse structure. Another reason may be the fact of not uniform dispersion of nanoparticles and formation of agglomerates in the microstructure of hardened cement paste.

### 3.3. Mechanical Properties

**Figure 8:**
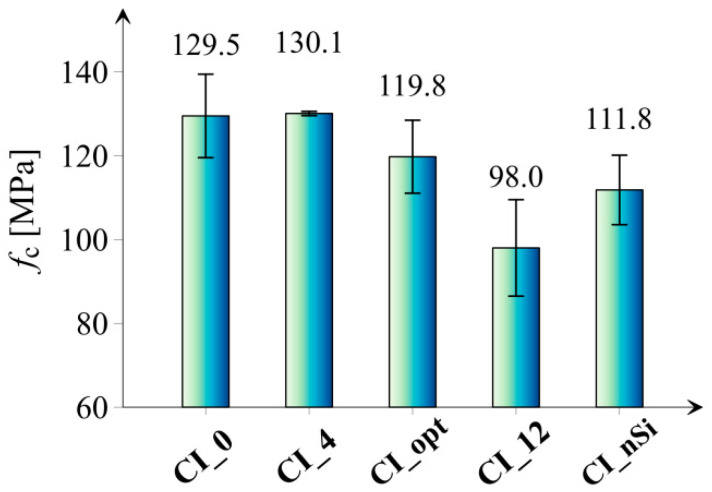
Results of compressive strength (f_c_) of all tested series. Vertical bars indicate one standard deviation.

Figure 8 presents the results of the compressive strength of all tested series.

**Figure 9:**
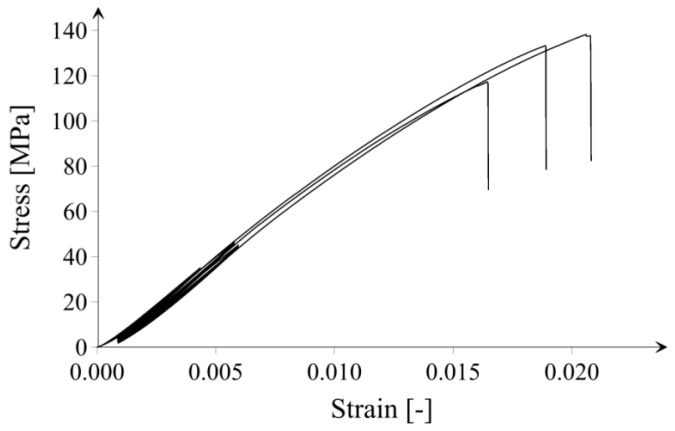
Course of the stress/strain curve from the compression test for three exemplary CI_0 samples.

Based on the results shown in Figure 8, it can be concluded that the addition of microsilica only in the amount of 4.0% resulted in a minimal enhancement in compressive strength in relation to the reference series; however, considering error bars, the difference is statistically negligible. In the case of the CI_opt series, a significantly lower value of compressive strength (approx. 7.5%) can be observed compared to the reference series, which is considered as an unexpected result, especially the study [22] that indicates the optimal content of microsilica in cement paste to be 10 wt. % in regard to compressive strength at 7, 28, 56 days. It is worth noticing that the application of microsilica in an amount greater than optimal causes a slight decrease in the compressive strength measured after 180 days of samples maturation. Lower compressive strength of series with the addition of silica fume (upon around 5%) compared to the reference series can be associated with the inhomogeneous distribution of microsilica in high amount or acting the excessive part of microsilica particles as just filler after the consumptions of portlandite by time. Note that comparatively high values of compressive strength for all series are the effect of a high degree of samples maturity, low water-to-solid ratio (0.36), as well as small sizes of investigated samples. A similar phenomenon was also observed in [45]. It was shown that the addition of microsilica in the cement paste improves the hydration kinetics of the cement, improving the compressive strength. However, with increasing the amount of microsilica additive, this effect disappears due to the strong agglomeration of the SF particles. Additionally, Figure 9 shows the course of the stress/strain curve from the compression test. The obtained curves confirm that the investigations of compressive strength characterized a good convergence, which proves the reliability of the obtained results.

### 3.4. Micromechanical Properties

**Figure 10:**
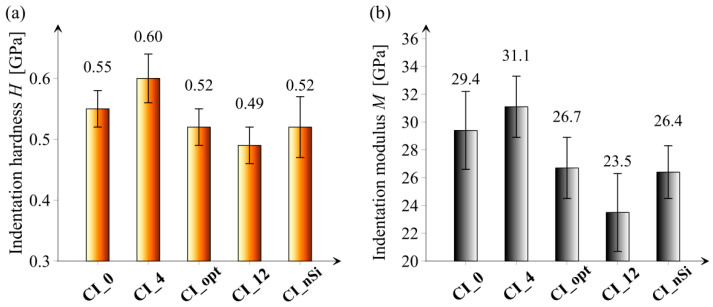
Results of: (**a**) hardness (H) and (**b**) indentation modulus (M) of all tested series. Vertical bars indicate one standard deviation.

Figure 10 presents the results of hardness and indentation modulus of all tested series.

When analyzing the hardness results, it can be noticed that the highest value was obtained for the CI_4 series, and the lowest for the CI_12 series. The other series CI_0, CI_opt, and CI_nSi obtained comparable hardness values. The test results of the indentation modulus reflect the hardness measurements. It is worth emphasizing that creating the optimal composition of cement pastes in regard to the content of microsilica and the planned effect of improving porosity does not result in excessive deterioration of micromechanical properties.

Since the beginning of the 21st century cementitious composites have been composed of more and more components, and proper design tools are needed to help in this process. However, since the simplified models and approaches are not enough yet, researchers still need to prepare large number of mixes with various additives proportions to find the optimum one. In addition, the more additives in the mix, the more combinations must be considered, which in turn implies a large number of tests that involve a lot of samples and used material.

**Figure 11:**
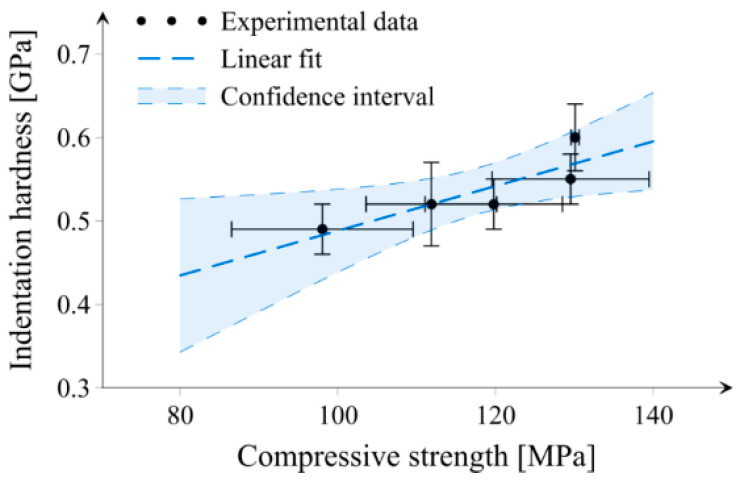
The relation between compressive strength and indentation hardness for all investigated sample series. Horizontal and vertical bars indicate one standard deviation.

One of the most commonly performed tests to verify the performance of cementitious composites is the compressive strength test. An alternative way to indirectly compare the strength performance for different cement paste mixes can be the use of the indentation technique, since a sufficient correlation between the compressive strength and indentation hardness of investigated sample series exists, which can be seen in Figure 11. It should be noted that the sample size for indentation is much smaller compared to samples needed for the compressive strength tests. In addition, the indentation technique can deliver information about elastic properties or creep as well [46].

### 3.5. Microstructural Analysis

**Figure 12:**
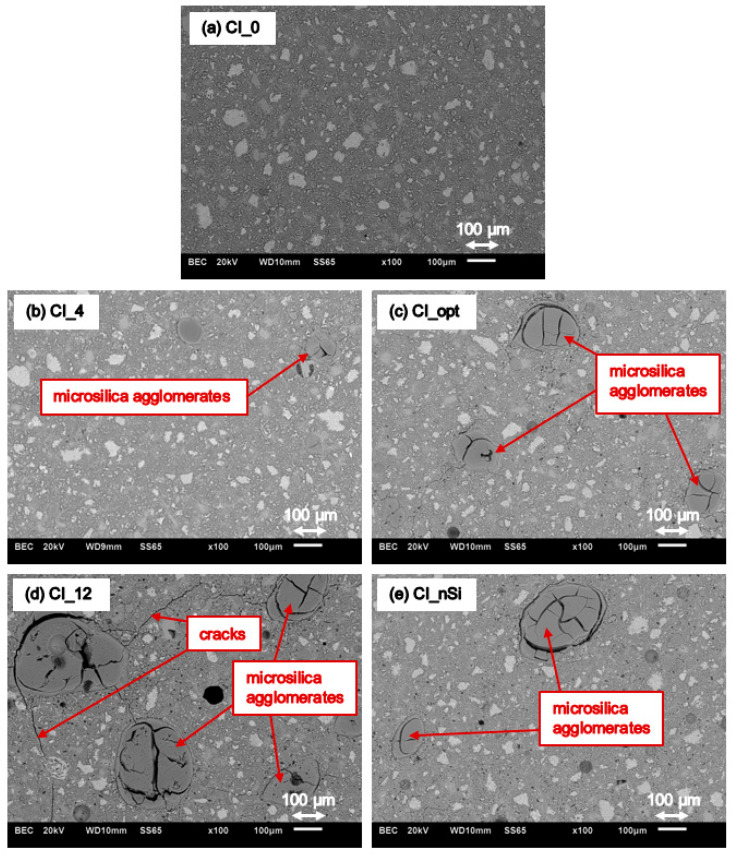
Microstructure of series: (**a**) CI_0, (**b**) CI_4, (**c**) CI_opt, (**d**) CI_12 and (**e**) CI_nSi.

Results of SEM investigations for each series of cement paste are presented in Figure 12.

Considering the above results, it should be said that the reference series CI_0 is characterized by a homogeneous microstructure, free of inclusions, cracks, or discontinuities (Figure 12a). On the other hand, in the series with the addition of microsilica, agglomerates of microsilica with a size greater than 100 µm can be seen, e.g., in Figure 12c. In the case of the series having the highest percentage of microsilica, i.e., CI_12, it is also seen that, in addition to the large number of agglomerates, cracks in the structure are clearly visible, as shown in Figure 12d. It seems that the presence of oversized microsilica may be an effect of an insufficient and non-effective mixing process, and in consequence, may cause deterioration of hardened cement paste structure in regard to its mechanical properties. The negative effect of agglomerated and oversized microsilica, acting as an aggregate rather than micro-size filler, was also observed in work [35].

**Figure 13:**
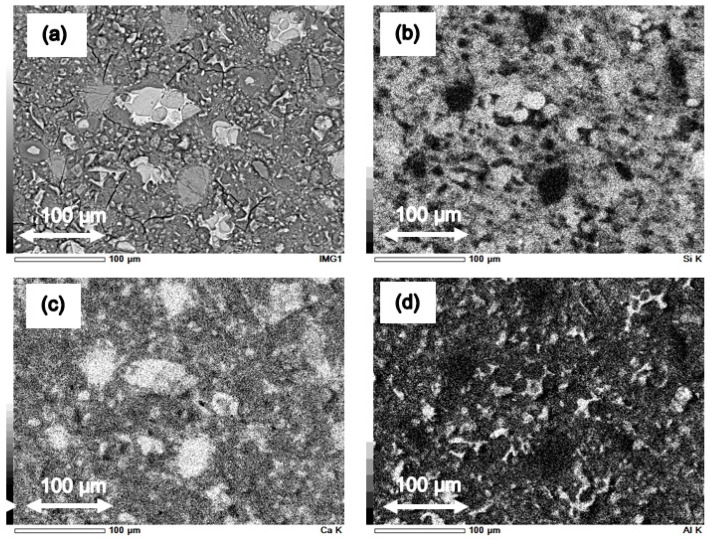
EDX mappings of chemical elements in the microstructure of the reference series CI_0: (**a**) area of the performed distribution, (**b**) images of Si incidence, (**c**) images of Ca incidence, (**d**) images of Al incidence.

**Figure 14:**
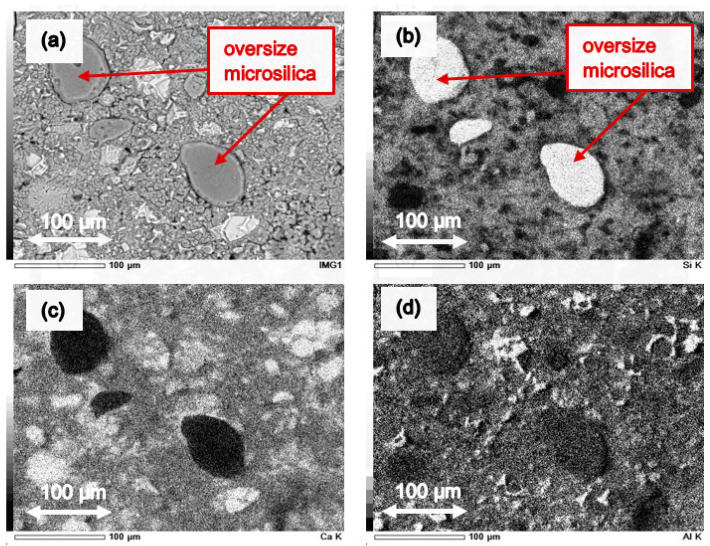
EDX mappings of chemical elements in the microstructure of the CI_opt series: (**a**) area of the performed distribution, (**b**) images of Si incidence, (**c**) images of Ca incidence, (**d**) images of Al incidence.

To confirm the presence of microsilica agglomerates in cement paste series with the addition of microsilica, EDX mappings with chemical elements distribution were performed. Thus, Figure 13 shows maps with chemical elements in the microstructure of the examined reference series CI_0, and Figure 14 presents maps with chemical elements in the microstructure of CI_opt cement paste series, respectively. It should be noted that in case of reference series CI_0 the microstructure is homogeneous (Figure 13a), and there are no agglomerates of microsilica (Figure 13b), while in the case of series CI_opt with the addition of almost 8% wt. of microsilica some inclusion of oversize silica fume are clearly visible (Figure 14a,b).

### 3.6. ASR

**Figure 15:**
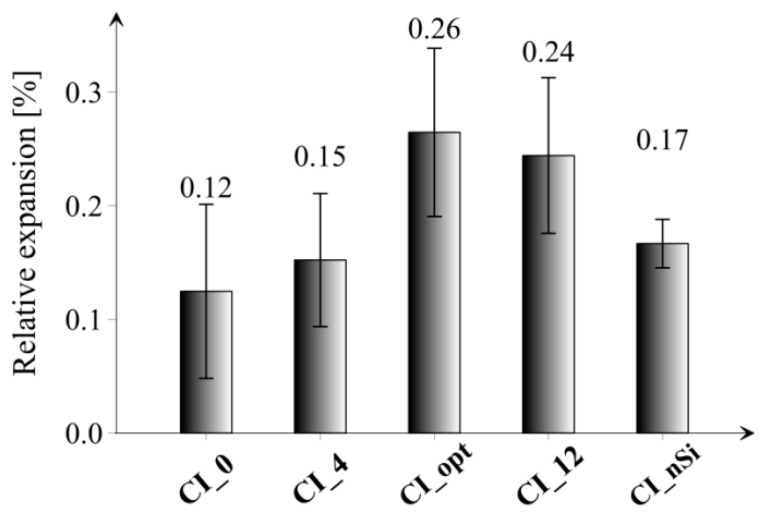
Results of ASR tests in the form of the relative increase in the length of the prismatic specimens of all tested series after 14 days of immersion in NaOH solution. Vertical bars indicate one standard deviation.

Figure 15 presents the results of ASR tests in the form of relative elongation of all tested series after 14 days of immersion in NaOH solution at heightened temperature.

Considering the results, it can be noticed that the smallest value of elongation was obtained for the reference series CI_0, and the largest for the series CI_opt and CI_12, i.e., the series with the highest content of microsilica in the composition. The study did not specify the minimum value of sample elongation, which would indicate the appearance of ASR; however, comparative analysis between results was performed. The elongation for the CI_opt series is at the level of 0.26%, which may be considered as a reliable value, especially referring it to the results of the tests described in paper [47]. On the other hand, in case of series with the addition of nanoparticles (CI_nSi), the ASR effect was not so clearly visible compared to the reference series. Another fact is that the standard deviation of the obtained results is quite large; hence, slight differences in the individual values of elongation for each of the tested series are acceptable. The observed elongation of samples may be related to the chemically and physically bound water in hydration products of microsilica and portlandite. In the surrounding of highly alkaline and hot water environment such high expansions can be expected due to the physically adsorbed water depending on the Ca/Si ratio of the matrix [28]. Moreover, microsilica agglomerates present in the series CI_4, CI_opt, CI_12 and CI_nSI (see Figure 12) can act as reactive aggregates rather than as pozzolans and thus cause expansion [35].

### 3.7. Discussion

The combination of the particle size distribution of individual components together with the modified Andreasen and Andersen packing density model allowed for the attainment of the optimized composition of the cement paste mixtures with the addition of only microsilica and microsilica with nanoparticles. As the amount of the microsilica additive in cement paste composition increases, both the bulk density and the specific density decreases. Cement paste with the optimized amount of microsilica is characterized by the lowest porosity, which may prove effectiveness of the packing density model used in the optimization process of cement paste mixes. However, the packing density model considers only the packing of all solids and does not take into account other factors, such as chemical reactivity of components, workability, water demand [22], etc., and therefore the lowest porosity did not reflect the highest mechanical properties, as it was presented by Chen et al. in [48] where metakaolin and silica fume were used in order to improve packing density and performance of binder paste. Notably, the combined application of the optimized amount of microsilica and nanoparticles did not bring the expected effect in the form of obtaining the lowest porosity. The reason for this may be the high consumption of batch water by nanoparticles (the effect of large specific surface area), as well as their irregular dispersion and formation of agglomerates in the microstructure of hardened cement paste.

Optimization of the composition of the cement paste with the use of AA packing density model did not increase the compressive strength (after 180 days of specimens’ maturation) nor the hardness, compared to the reference series and the series with the addition of 4 wt. % microsilica. The reason of that fact can be presence of microsilica agglomerates, which has been confirmed by microstructural analysis, and what is probably a consequence of ineffective mixing process. Moreover, oversize microsilica could act as reactive aggregate and cause alkali-silica reaction. A phenomenon in which microsilica induces instead of inhibiting the ASR reaction is a kind of paradox, described and explained in detail in paper [35]. The described mechanism might deteriorate microstructure by crack appearance and lower compressive strength. It should be noted here that working on paste level can also be a reason for undispersed microsilica due to the lack of the internal shearing effect of aggregates during mixing. Nevertheless, creating the optimal composition of cement pastes regarding the content of microsilica and the planned effect of improving porosity did not result in excessive deterioration of mechanical properties. At this point, it is worth emphasizing that the application of microsilica as a partial replacement for cement meets the idea of sustainable development and encourages an attempt to limit CO_2_ emissions. Hence, even with no significant improvement of the mechanical properties of the cement paste with the addition of microsilica, its use may be considered advantageous, considering at least the environmental conditions, together with the lower density and lower porosity.

## 4. Conclusions

Based on the above, the following conclusions were drawn that can be used as practical guidance for researchers and engineers.

- The packing density model is an effective tool in the case of designing the cement paste with the addition of the optimal amount of microsilica regarding the lowest porosity. In this case, the use of about 7.5 wt. % of microsilica is optimum. Note that this value depends on the type of microsilica and cement characterized by different particle size distributions.
- On the other hand, if cement paste is designed, mainly in regard to mechanical properties, such as compressive strength and hardness, then the use of the packing density model does not bring satisfactory results. To improve the mechanical properties of cement paste, the use of 4 wt. % of microsilica appeared to be optimal, which value is not a result of applied AA packing density model.
- In the case of using microsilica as an additive to cement paste, the mixing process is an important issue. It may be related with inhomogeneous dispersion of microsilica and creation of agglomerates acting as reactive aggregates that in consequence cause ASR reaction, crack occurrence, and lowered values of mechanical properties.

It must be emphasized that the presented results are part of the first stage of the research project aimed at determining the optimal amount of microsilica and SiO_2_ nanoparticles in cement-based materials with the use of the packing density model and in regard to the selected physical and mechanical properties of the obtained composite. In the next stage, on the basis of the obtained results, it is planned to investigate the effectiveness of the application of the packing density model at the mortar and concrete level.

## Figures and Tables

**Table 1:** Chemical and phase composition of Portland cement CEM I 42.5R.

Component	[wt.%]	Phase	[wt.%]
CaO	66.00	Tricalcium silicate (C_3_S)	56.46
SiO_2_	21.50	Dicalcium silicate (C_2_S)	19.29
Al_2_O_3_	5.60	Tricalcium aluminate (C_3_A)	10.11
Fe_2_O_3_	2.80	Tetracalcium aluminoferrite (C_4_AF)	8.51
MgO	1.50	Gypsum (CSH_2_)	5.38
SO_3_	2.50		
Cl^−^	0.08		
Loss of ignition	3.30		

**Table 2:** Chemical composition of microsilica 920 EN D.

Component	[wt.%]
SiO_2_	≥85.00
SO_3_	<2.00
Cl^−^	<0.30
Free CaO	<1.00
Free Si	<0.40
Loss of ignition	<4.00

**Table 3:** Composition of the designed cement paste mixes used in tests.

Cement Paste	Microsilica [kg/m^3^] (wt.%)	Nanoparticles [kg/m^3^] (wt.%)	Cement [kg/m^3^]	Water [kg/m^3^]	SP [kg/m^3^]
CI_0	–	–	1463	527	15
CI_4	55 (4.00%)	–	1387	519	14
CI_opt	101 (7.63%)	–	1325	514	13
CI_12	151 (12.00%)	–	1258	507	13
CI_nSi	120 (9.34%)	12 (0.93%)	1286	511	13

## Data Availability

The data presented in this study are available on request from the corresponding author.
